# Supplementary figures and images for: Harnessing AI and analytics to enhance cybersecurity and privacy for collective intelligence systems
Source: PeerJ Comput Sci. 2024 Sep 20;10:e2264. doi: 10.7717/peerj-cs.2264 (PMC11419604; doi:10.7717/peerj-cs.2264)

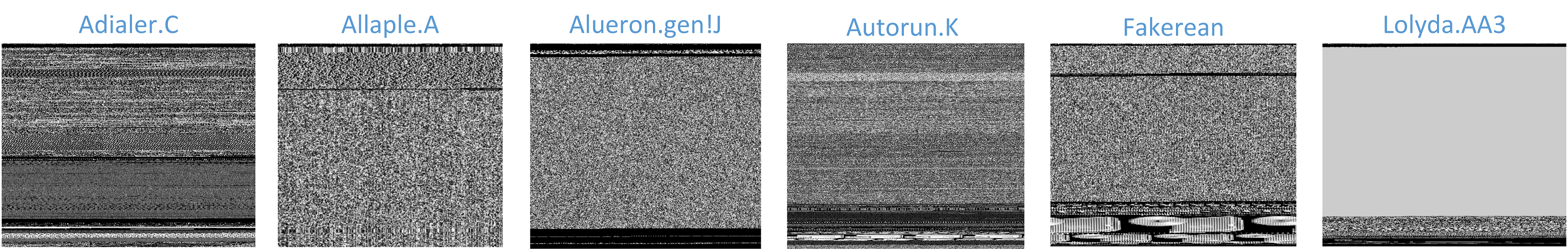

Supplement: Supplemental Information 2 [file peerj-cs-10-2264-s002.png]

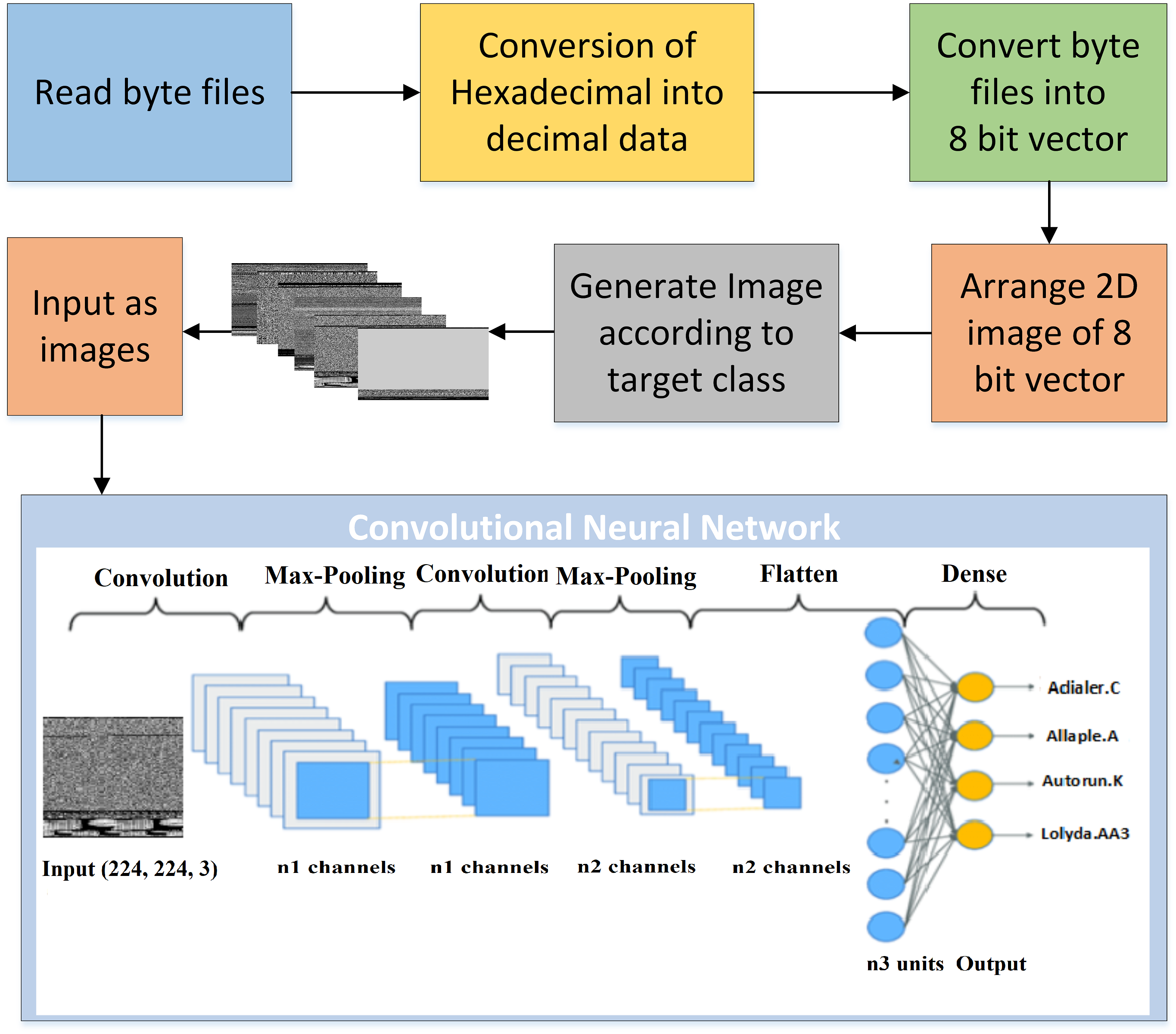

Supplement: Supplemental Information 3 [file peerj-cs-10-2264-s003.png]

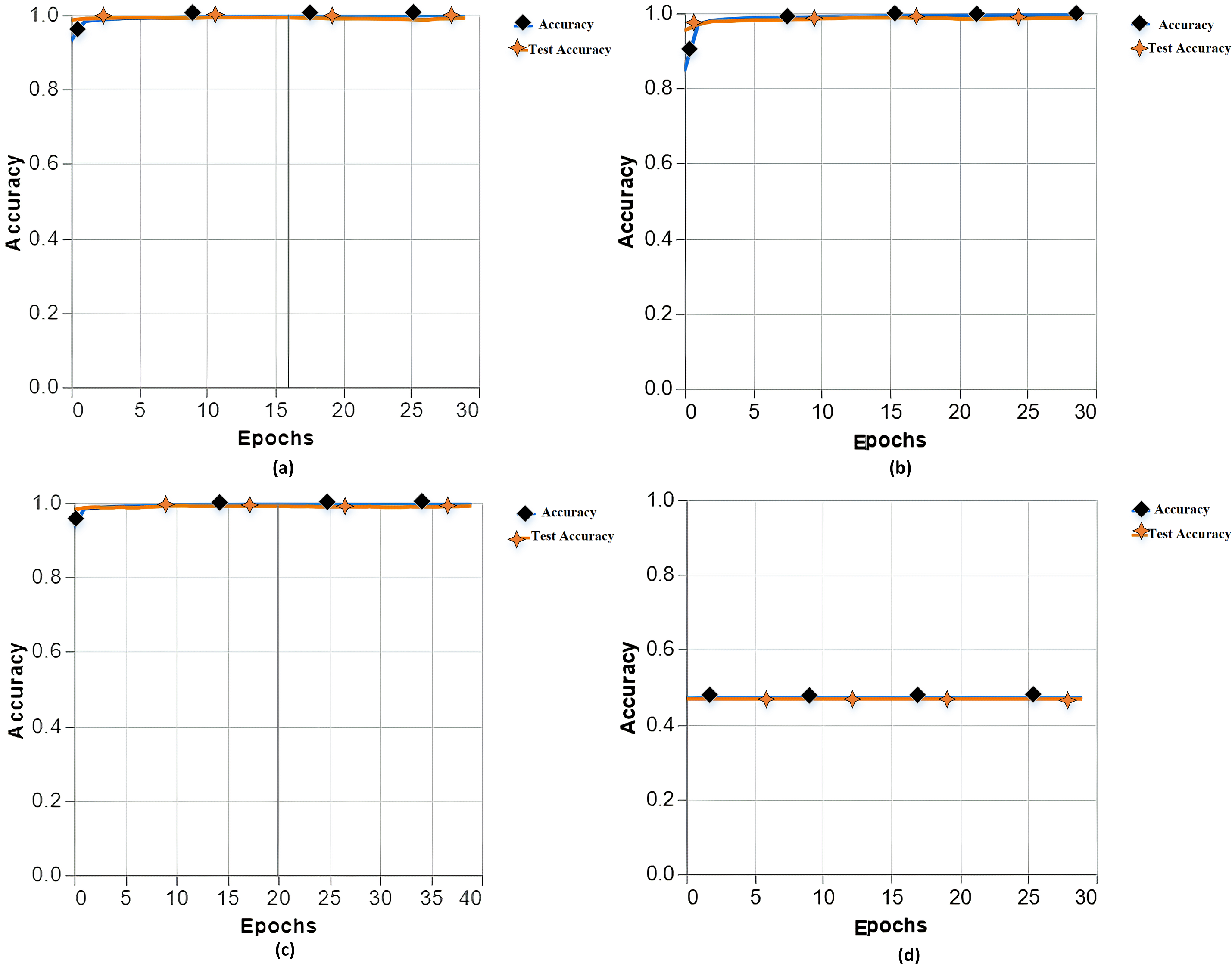

Supplement: Supplemental Information 4 [file peerj-cs-10-2264-s004.png]

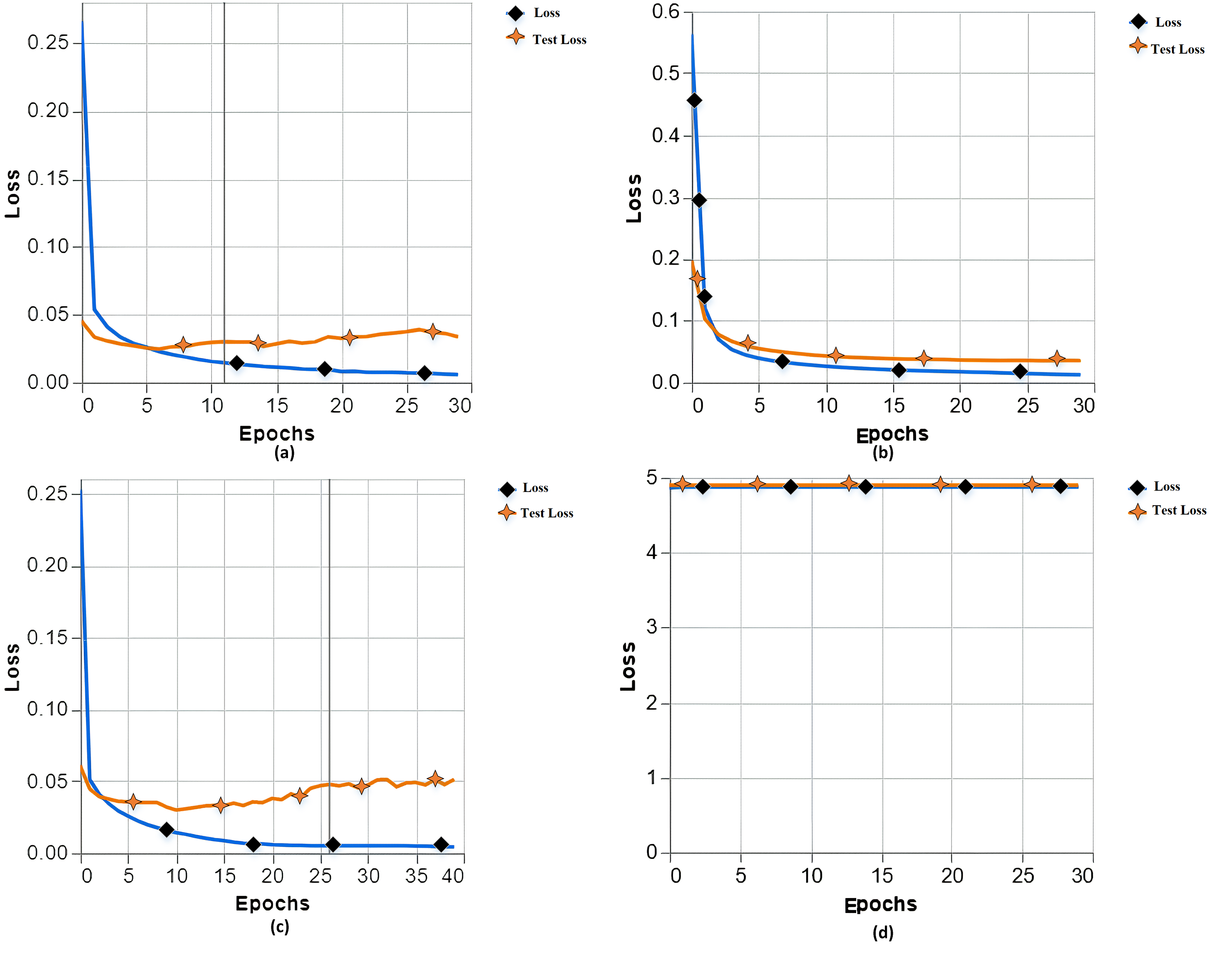

Supplement: Supplemental Information 5 [file peerj-cs-10-2264-s005.png]

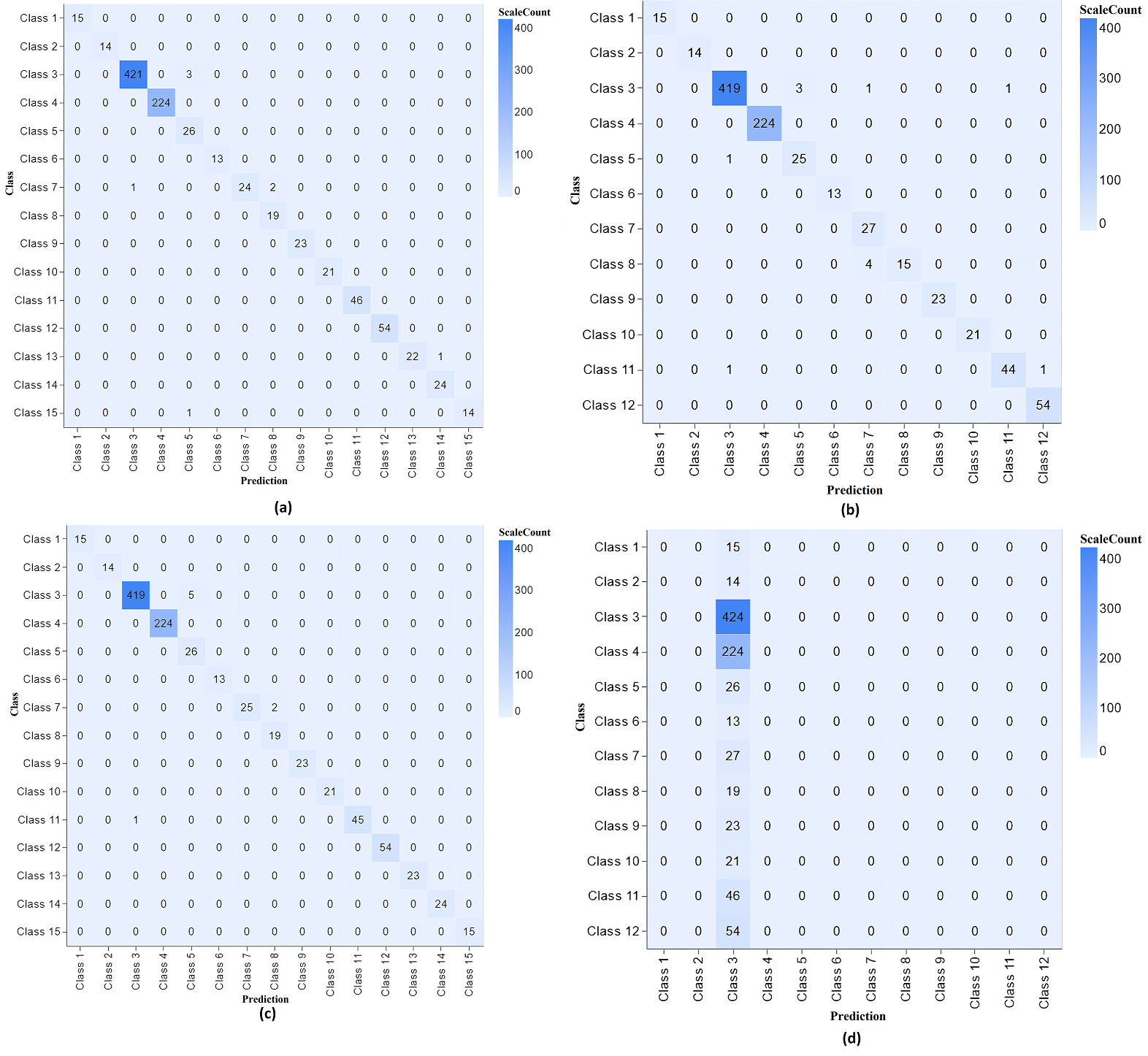

Supplement: Supplemental Information 6 [file peerj-cs-10-2264-s006.png]

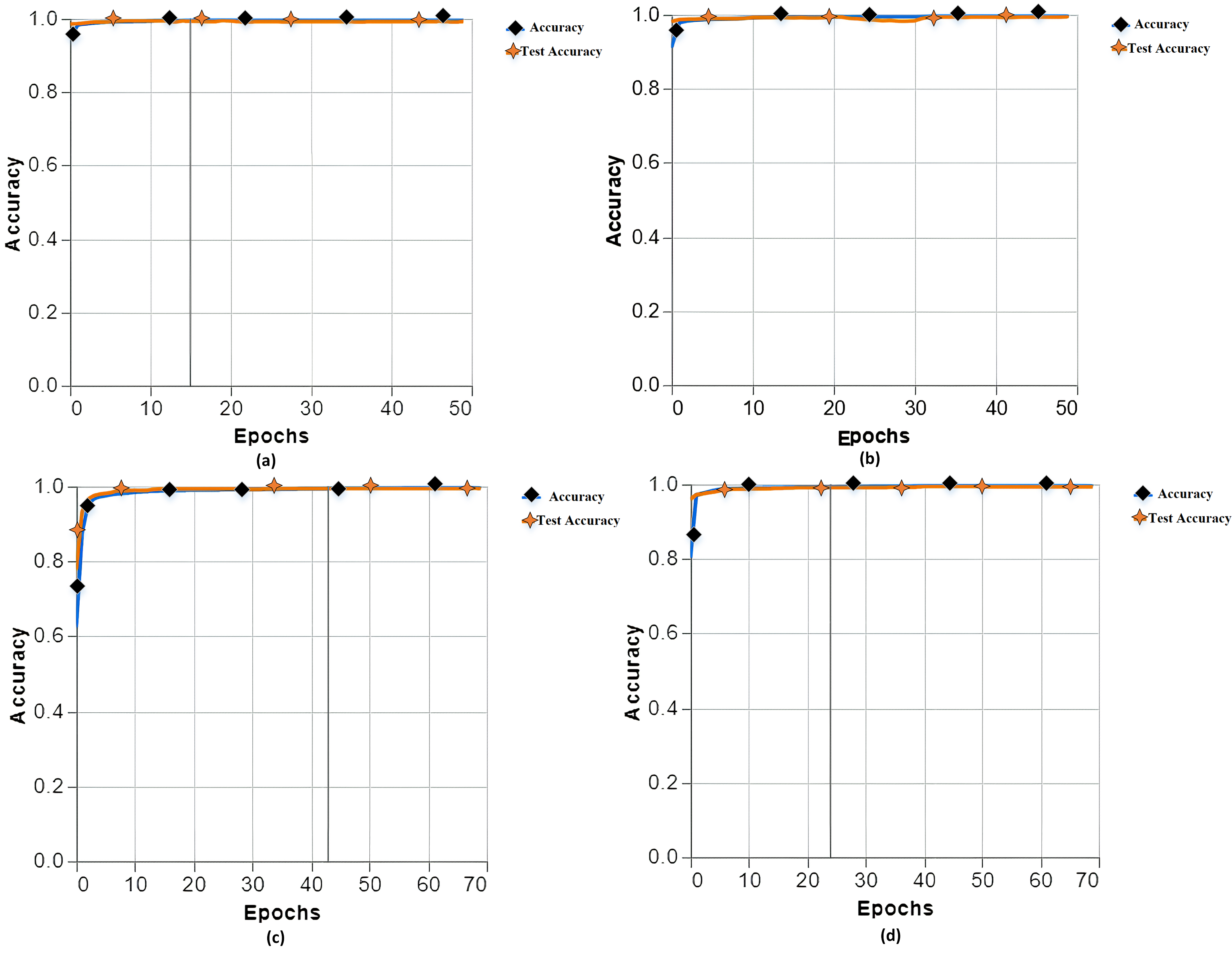

Supplement: Supplemental Information 7 [file peerj-cs-10-2264-s007.png]

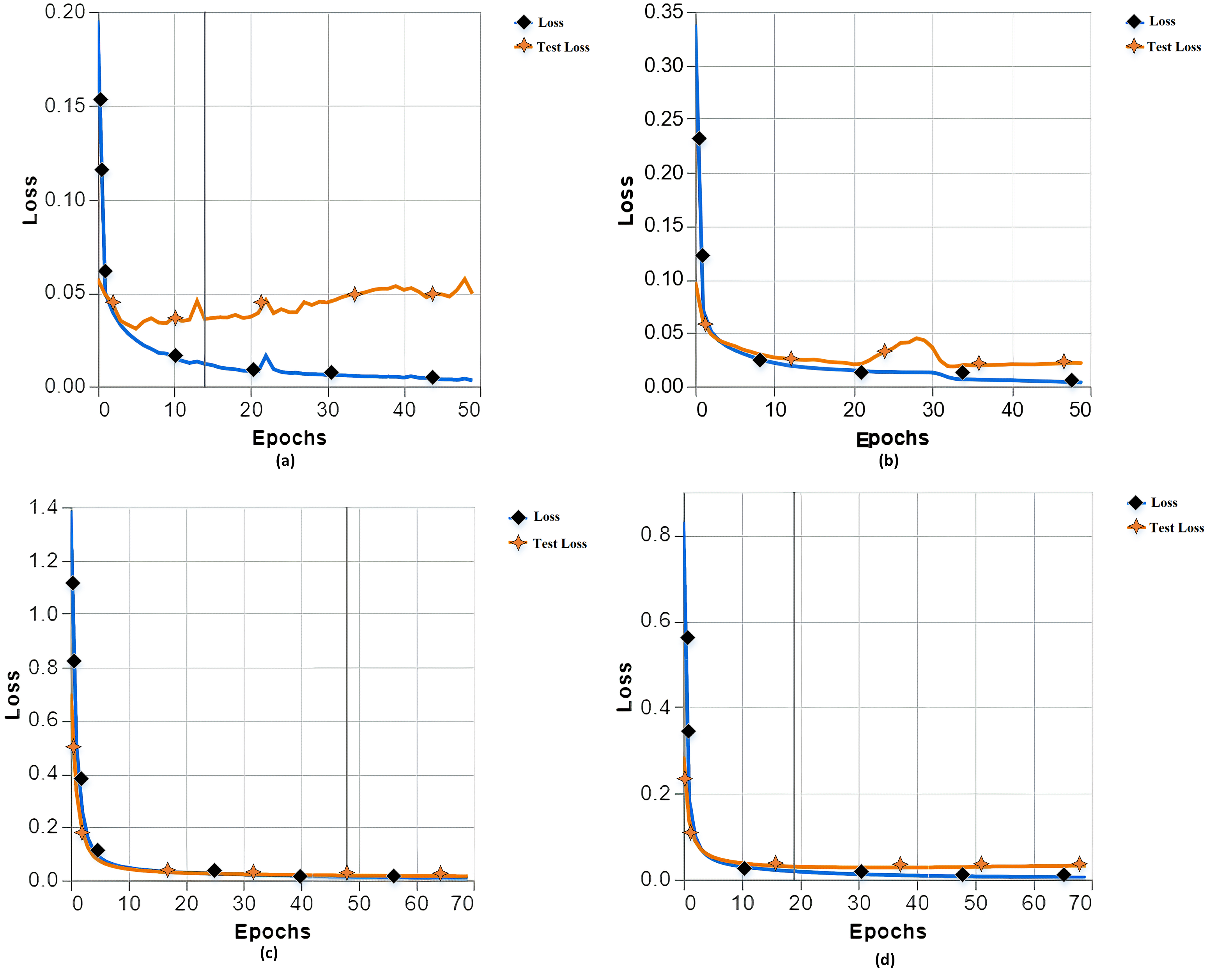

Supplement: Supplemental Information 8 [file peerj-cs-10-2264-s008.png]

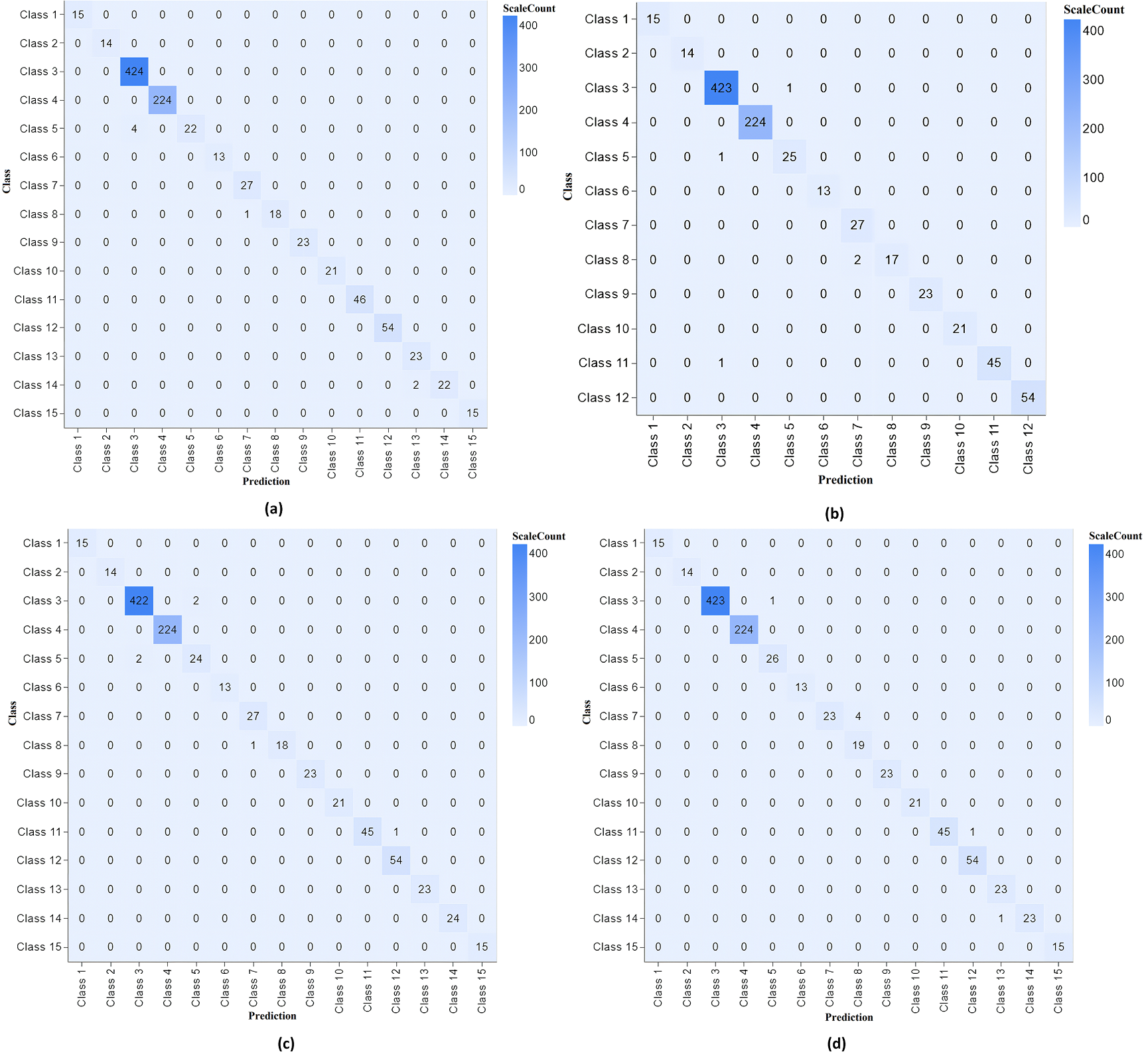

Supplement: Supplemental Information 9 [file peerj-cs-10-2264-s009.png]
