# Supplementary material for: Harnessing AI and analytics to enhance cybersecurity and privacy for collective intelligence systems
Source: PeerJ Comput Sci. 2024 Sep 20;10:e2264. doi: 10.7717/peerj-cs.2264 (PMC11419604; doi:10.7717/peerj-cs.2264)
Supplement: Supplemental Information 12 [file peerj-cs-10-2264-s012.docx]

| **Epoch** | **Batch size** | **Test accuracy** | **Test loss** |
| --- | --- | --- | --- |
| 30 | 16 | 46.79 | 4.9 |
| 25 | 116 | 98.65 | 0.0476 |
| 30 | 128 | 98.67 | 0.0356 |
| 20 | 64 | 98.86 | 0.0402 |
| 30 | 32 | 99.17 | 0.0337 |
| 40 | 32 | 99.17 | 0.0516 |
| 70 | 16 | 99.27 | 0.0321 |
| 50 | 16 | 99.27 | 0.0494 |
| 70 | 512 | 99.38 | 0.0158 |
| 50 | 64 | 99.44 | 0.0217 |
| 20 | 16 | 99.48 | 0.0214 |
| 20 | 64 | 99.56 | 0.0378 |
| 70 | 16 | 99.86 | 0.0558 |

Table 7: Assessment of statistical significance of Test accuracy and Test loss.
