# Supplementary material for: Harnessing AI and analytics to enhance cybersecurity and privacy for collective intelligence systems
Source: PeerJ Comput Sci. 2024 Sep 20;10:e2264. doi: 10.7717/peerj-cs.2264 (PMC11419604; doi:10.7717/peerj-cs.2264)
Supplement: Supplemental Information 13 [file peerj-cs-10-2264-s013.docx]

| **Epoch** | **Batch size** | **Learning rate** | **Loss** |
| --- | --- | --- | --- |
| 30 | 16 | 0.1 | 4.87 |
| 25 | 116 | 0.001 | 0.0066 |
| 30 | 128 | 0.001 | 0.0127 |
| 20 | 64 | 0.0001 | 0.0364 |
| 30 | 32 | 0.001 | 0.0059 |
| 40 | 32 | 0.001 | 0.0046 |
| 70 | 16 | 0.0001 | 0.0051 |
| 50 | 16 | 0.001 | 0.0031 |
| 70 | 512 | 0.001 | 0.0084 |
| 50 | 64 | 0.001 | 0.0037 |
| 20 | 16 | 0.001 | 0.0065 |
| 20 | 64 | 0.001 | 0.012 |
| 70 | 16 | 0.001 | 0.0103 |

Table 9: Measuring statistical significance of Learning rate and Loss.
