# Supplementary material for: Harnessing AI and analytics to enhance cybersecurity and privacy for collective intelligence systems
Source: PeerJ Comput Sci. 2024 Sep 20;10:e2264. doi: 10.7717/peerj-cs.2264 (PMC11419604; doi:10.7717/peerj-cs.2264)
Supplement: Supplemental Information 14 [file peerj-cs-10-2264-s014.docx]

| **Epoch** | **Batch size** | **Learning rate** | **Accuracy** |
| --- | --- | --- | --- |
| 30 | 16 | 0.1 | 47.07 |
| 25 | 116 | 0.001 | 99.74 |
| 30 | 128 | 0.001 | 99.52 |
| 20 | 64 | 0.0001 | 98.84 |
| 30 | 32 | 0.001 | 99.72 |
| 40 | 32 | 0.001 | 99.76 |
| 70 | 16 | 0.0001 | 99.79 |
| 50 | 16 | 0.001 | 99.87 |
| 70 | 512 | 0.001 | 99.77 |
| 50 | 64 | 0.001 | 99.92 |
| 20 | 16 | 0.001 | 99.76 |
| 20 | 64 | 0.001 | 99.6 |
| 70 | 16 | 0.001 | 99.81 |

Table 10: Statistical significance testing of Learning rate and Accuracy.
