# Supplementary material for: Harnessing AI and analytics to enhance cybersecurity and privacy for collective intelligence systems
Source: PeerJ Comput Sci. 2024 Sep 20;10:e2264. doi: 10.7717/peerj-cs.2264 (PMC11419604; doi:10.7717/peerj-cs.2264)
Supplement: Supplemental Information 15 [file peerj-cs-10-2264-s015.docx]

Dear Editor,

Thank you for considering our revised manuscript titled “Harnessing AI and analytics to enhance cybersecurity and privacy for collective intelligence systems” for publication in PeerJ. In response to the peer review comments requesting more details on author contributions, please find below a detailed account of the intellectual and practical contributions made by the proposed additional authors:

Faiz Abdullah Alotaibi substantially improved the overall quality of our paper. He enhanced the readability and logical flow of the text by refining sentences for clarity and correcting grammatical errors. Mr. Alotaibi also played a significant role in revising the manuscript in response to the reviewers’ feedback.

Gabriel Avelino Sampedro conducted novel experimental work presented in the revised manuscript. In particular, he enhanced key figures through advanced image processing techniques, making results more informative and accurately depicting changes in accuracy and test accuracy. Furthermore, Mr. Sampedro refined experimental methodology and description to improve comprehension for scientific audiences.

Sample of the previous image:

Sample of revised image:

Vincent Karovič additionally performed experimental work integral to revisions. He similarly improved figures through enhanced visualization of loss and test loss. Mr. Karovič assisted significantly in revising the manuscript to address reviewer comments.

Sample of the previous image:

Sample of revised image:

We hope this detailed accounting of intellectual and practical contributions from each proposed additional author sufficiently demonstrates their merit for inclusion in the authorship. Please do not hesitate to request any further information. We appreciate your time and consideration.

Sincerely,

Muhammad Rehan Naeem

Corresponding Author
