# Supplementary material for: Harnessing AI and analytics to enhance cybersecurity and privacy for collective intelligence systems
Source: PeerJ Comput Sci. 2024 Sep 20;10:e2264. doi: 10.7717/peerj-cs.2264 (PMC11419604; doi:10.7717/peerj-cs.2264)
Supplement: Supplemental Information 16 [file peerj-cs-10-2264-s016.docx]

Dear Editor,

Thank you for handling our manuscript submitted to PeerJ. Upon further review of the author's contributions, we found an error in our initial submission that requires correction.

Nabil Sharaf Almalki was incorrectly included as an author in the original manuscript. While Mr. Almalki has collaborated productively with our research group on other projects, he did not contribute substantively to this work.

When we began developing this study, Mr. Almalki was initially involved but withdrew at an early stage due to other commitments. As such, he performed no experimental work, data analysis, or writing related to the current submission.

Regrettably, his name was inadvertently retained in the author's byline when we submitted the manuscript despite lacking intellectual or practical contributions. I sincerely apologize for this oversight on our part.

Given that Mr. Almalki made no material contribution to the research presented, we formally request that his name be removed from the author list (mentioned in early submission in Confidential Information for PeerJ Staff). He has acknowledged and consented to this correction.

Please accept my sincerest apologies for the erroneous inclusion of the author. I hope we can rectify this issue and move forward with peer review. Please let me know if any other information would be helpful.

Thank you in advance for your understanding.

Sincerely,

Muhammad Rehan Naeem

Corresponding Author
